# Conflicting views of physicians and surgeons concerning pediatric urinary tract infection: a comparative review

**DOI:** 10.1007/s00247-023-05771-x

**Published:** 2023-09-30

**Authors:** Ian K. Hewitt, Derek J. Roebuck, Giovanni Montini

**Affiliations:** 1 Department of Pediatric Nephrology, Perth Children’s Hospital, Nedlands, 6009 Australia; 2 Division of Pediatrics, Medical School, University of Western Australia, Crawley, 6009 Australia; 3 Department of Medical Imaging, Perth Children’s Hospital, Nedlands, 6009 Australia; 4 Pediatric Nephrology, Dialysis and Transplant Unit, Fondazione Ca’ Granda IRCCS, Policlinico di Milano, Milan, Italy; 5 Giuliana and Bernardo Caprotti Chair of Pediatrics, Department of Clinical Sciences and Community Health, University of Milano, Milan, Italy

**Keywords:** Antibiotic prophylaxis, Cystography, Pediatric, Review, Urinary tract infections, Vesico-ureteral reflux

## Abstract

**Background:**

A first febrile urinary tract infection (UTI) is a common condition in children, and pathways of management have evolved over time.

**Objective:**

To determine the extent to which pediatricians and surgeons differ in their investigation and management of a first febrile UTI, and to evaluate the justifications for any divergence of approach.

**Materials and methods:**

A literature search was conducted for papers addressing investigation and/or management following a first febrile UTI in children published between 2011 and 2021. Searches were conducted on Medline, Embase, and the Cochrane Controlled Trials Register. To be eligible for inclusion, a paper was required to provide recommendations on one or more of the following: ultrasound (US) and voiding cystourethrogram (VCUG), the need for continuous antibiotic prophylaxis and surgery when vesicoureteral reflux (VUR) was detected. The authorship required at least one pediatrician or surgeon. Authorship was categorized as medical, surgical, or combined.

**Results:**

Pediatricians advocated less imaging and intervention and were more inclined to adopt a “watchful-waiting” approach, confident that any significant abnormality, grades IV–V VUR in particular, should be detected following a second febrile UTI. In contrast, surgeons were more likely to recommend imaging to detect VUR (*p*<0.00001), and antibiotic prophylaxis (*p*<0.001) and/or surgical correction (*p*=0.004) if it was detected, concerned that any delay in diagnosis and treatment could place the child at risk of kidney damage. Papers with combined authorship displayed intermediate results.

**Conclusion:**

There are two distinct directions in the literature regarding the investigation of an uncomplicated first febrile UTI in a child. In general, when presented with a first febrile UTI in a child, physicians recommend fewer investigations and less treatment, in contrast to surgeons who advocate extensive investigation and aggressive intervention in the event that imaging detects an abnormality. This has the potential to confuse the carers of affected children.

**Supplementary Information:**

Supplementary material is available at 10.1007/s00247-023-05771-x.

## Introduction

Febrile urinary tract infections (UTIs) are common in children. In infants presenting with unexplained fever the prevalence of UTI is 7%, reaching 20% in uncircumcised boys by three months of age [1]. These febrile UTIs are said to lead to pyelonephritic scarring in up to 30% of cases, and can be the first sign of a congenital abnormality of the kidney and urinary tract, the most frequent being vesicoureteral reflux (VUR), which occurs in one-third of cases [2]. The observation that VUR is a risk factor for recurrent infection, and the finding of an association between VUR (primarily high-grade) and chronic kidney damage, originally led to a push from the medical and surgical world to try to detect VUR after a febrile UTI, with the implementation of continuous antibiotic prophylaxis or surgical correction in the event of finding it. In recent years, there has been a reassessment of the role of VUR and acquired pyelonephritic scarring as risk factors for progressive chronic kidney disease and other long-term consequences [3, 4], and thus of the need to investigate children following a first febrile UTI.

The role of the pediatric radiologist is to decide whether a voiding cystourethrogram (VCUG), the standard test for VUR, is justified in an individual patient, and if so to perform and interpret it. A first febrile UTI in a young child can present to a pediatrician or a pediatric surgeon.

We are aware of an interesting paper that appeared in the BMJ some years ago entitled “Phenotypic differences between male physicians, surgeons and film stars: comparative study,” where evidence-based medicine was contrasted with confidence-based medicine [5]. A further paper by Stirrat also provided an insight into surgical thinking [6]. In multidisciplinary discussions among radiologists, physicians, and surgeons at our institutions, it has become apparent that stark differences have developed in the medical and surgical approaches to investigation and management of this common condition.

To what extent are differences in approach to febrile UTIs present in the literature, and if so, what are the justifications? To answer these questions, we reviewed the literature, comparing the approaches of physicians and surgeons to investigation and treatment.

## Methods

**Fig. 1:**
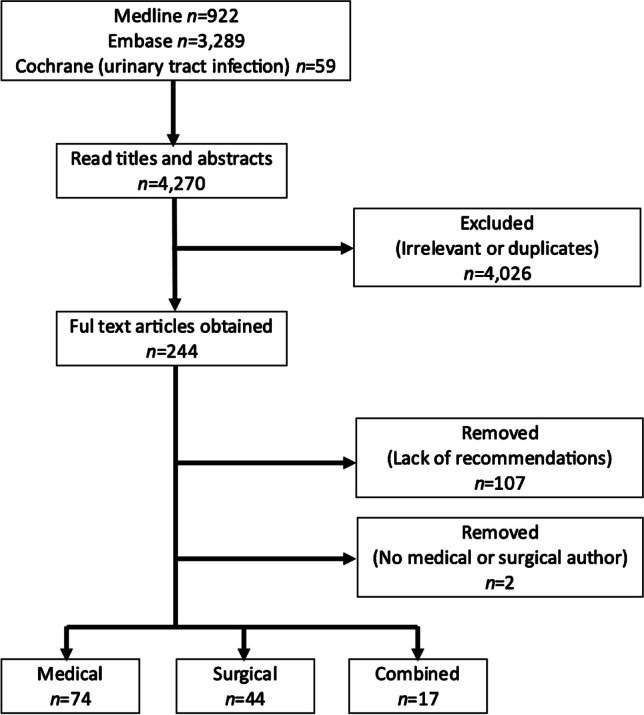
PRISMA flowchart

Searches were conducted on Medline, Embase, and the Cochrane Controlled Trials Register for reviews and studies in English, where the authors might make recommendations on the investigation and/or management of UTIs in children published between January 2011, the year of the revised American Academy of Pediatrics (AAP) recommendations [7] and December 2021. The websites of professional societies were also accessed for their guidelines, if available, on the investigation and management of a first febrile UTI in children. The Medline search strategy is included in Supplementary Material 1. The search strategy was adapted to the syntax and subject headings of Embase and the Cochrane Controlled Trials Register. To be eligible for inclusion, a paper was required to provide recommendations on one or more of the following: imaging in the form of ultrasound (US) and voiding cystourethrogram (VCUG), the need for continuous antibiotic prophylaxis, and surgery when VUR was detected. ^99m^Tc-dimercaptosuccinic acid scans are not commonly used after a first febrile UTI and were thus not included. All titles were reviewed, duplicates removed, abstracts read, and the full texts of potential articles obtained. The study selection was performed independently by two of the authors of this manuscript (I.K.H. and G.M., both with over 30 years of experience in pediatric nephrology) based on titles and abstracts; non-English language papers were excluded at this stage. Disagreement in selection and full-text review was resolved by consensus. One hundred and thirty-five papers, addressing investigation and/or management following a first febrile UTI, were selected for inclusion (Fig. 1).

The papers were divided into reviews and studies, with authorship classified as medical, surgical, or combined, according to author status and affiliations recorded in the articles. Where information was inadequate, authors were searched within their affiliated institutions on the internet. Statistical analysis was performed using the chi-squared test for comparison of proportions, with *P*-values <0.05 considered significant. The extended Cochran-Armitage test was used to evaluate the association between recommendation and authorship groups (surgeons, physicians, combined). Statistical analysis was performed using the open-source statistical software R [8].

## Results

Where recommendations on imaging have been made, ultrasound, being a non-invasive procedure without a radiation burden, is universally recommended following a first febrile UTI by physicians and surgeons alike, apart from the Caring for Australian and New Zealanders with Kidney Impairment (CARI) guidelines that recommend the test only in specific circumstances (Table 1). Most studies and reviews conducted by physicians, in contrast with surgeons, do not support performance of a VCUG following a first febrile UTI, or continuous antibiotic prophylaxis and surgery in the event VUR, particularly of milder grades, is found. The differences are statistically significant in all cases (Table 2). The papers with combined authors demonstrate intermediate results between physicians and surgeons, with the results closer to those of physicians. Including the “both” category in the analysis and considering the variables as ordered (surgeons>both>physicians), the association with all three outcomes is highly significant (extended Cochran-Armitage test): recommending VCUG: *P*<0.0001, recommending continuous antibiotic prophylaxis: *P*<0.001, recommending surgery: *P*=0.003.

**Table 1:** Summary of published recommendations for the investigation and management of a first febrile urinary tract infection, categorized according to authorship (2011 to 2021)

		Recommending VCUG	Recommending continuous antibiotic prophylaxis	Recommending surgery
Reviews	Physicians	5% (2/41)	10% (4/41)	8% (1/12)
	Surgeons	64% (7/11)	46% (6/13)	44% (4/9)
	Both	0% (0/8)	0% (0/6)	0% (0/3)
Studies	Physicians	24% (8/34)	22% (2/9)	25% (1/4)
	Surgeons	80% (20/25)	57% (4/7)	78% (7/9)
	Both	57% (4/7)	50% (1/2)	50% (1/2)
Reviews and studies	Physicians	13% (10/75)	12% (6/50)	13% (2/16)
	Surgeons	75% (27/36)	50% (10/20)	61% (11/18)
	Both	27% (4/15)	13% (1/8)	20% (1/5)

*VCUG* voiding cystourethrogram

**Table 2:** Comparison between recommendations published by physicians and surgeons

	Recommending VCUG	Recommending continuous antibiotic prophylaxis	Recommending surgery
Reviews	*χ*^2^ 20.92 df1 *P*<0.00001	*χ*^2^ 12.61 df1 *P*<0.001	*χ*^2^ 3.70 df1 *P*=0.055
Studies	*χ*^2^ 18.43 df1* P*<0.0001	*χ*^2^ 2.05 df1 *P*=0.15	*χ*^2^ 3.26 df1 *P*<0.07
Reviews and studies	*χ*^2^ 41.63 df1 *P*<0.00001	*χ*^2^ 11.70 df1 *P*<0.001	*χ*^2^ 8.48 df1 *P*=0.004

*VCUG* voiding cystourethrogram

### Reviews against imaging and interventions

Of the 51 reviews that support a reduction in VCUGs following a first febrile UTI, 39 are medical [4, 9–46], four surgical [47–50], eight combined [2, 51–57], and 45 are reference medical guidelines. The six reviews that do not, note the minimal benefit of interventions in reducing febrile UTI (including no reduction in scarring) and the opportunity to reduce cost, radiation exposure, and stress for the child and family [23, 27, 40, 44, 47, 50].

The 50 reviews against continuous antibiotic prophylaxis in the event of detection of VUR (37 medical [4, 9–11, 13, 15–25, 27–30, 32–38, 40, 41, 45, 46, 58–63], seven surgical [47–50, 64–66], six combined [51, 53, 56, 57, 67, 68]) variously cited National Institute for Health and Care Excellence and American Academy of Pediatrics guidelines, Cochrane, a range of randomized controlled trials (RCTs), and a long-term Swedish follow-up study [69]. Of note, the low incidence of recurrent febrile UTIs and scarring even in the control untreated groups led to questioning the value of any intervention [39].

Those against surgical intervention (11 medical [4, 9, 11, 13, 16, 17, 21, 29, 40, 59, 70], five surgical [47–50, 64], three combined [53, 56, 57]) cited meta-analyses and Cochrane reviews showing minimal benefit or benefit no better than continuous antibiotic prophylaxis.

### Reviews for voiding cystourethrograms and interventions

Of the 11 reviews for imaging with VCUGs, eight were surgical, citing the European Association of Urology - European Society for Paediatric Urology (EAU-ESPU) and/or American Urological Association (AUA) guidelines [71, 72], outdated American Academy of Pediatrics 1999 guidelines [73], concerns that delaying a VCUG until after a second febrile UTI placed children at risk of significant scarring [74], the high incidence of VUR associated with a febrile UTI [75], poor compliance with prophylaxis [31], concerns that antibiotic prophylaxis promotes drug resistance [76], and other urologists who support intervention [50]. The three medical reviews for imaging with VCUGs referred to the outdated American Academy of Pediatrics 1999 guidelines [77], Cincinnati guidelines [60], and Indian guidelines [78].

Of the ten reviews for continuous antibiotic prophylaxis, six were surgical, three referenced the RIVUR trial [2] as supportive of continuous antibiotic prophylaxis [79–81], one acknowledged that continuous antibiotic prophylaxis was of questionable value but best “err on the side of caution” [82], one referenced the American Urological Association guideline [71], and one cited increasing antibiotic resistance as a reason to consider surgery as a first line treatment [65]. The four medical reviews for continuous antibiotic prophylaxis cited the outdated American Academy of Pediatrics 1999 guidelines [43, 77, 78] and the RIVUR study demonstrating some benefit [42].

All four reviews for surgical intervention were authored by surgeons. They cited the American Urological Association guidelines, the PRIVENT and Swedish Reflux trials [71, 75, 81], and concerns regarding increased antibiotic resistance with continuous antibiotic prophylaxis [65].

### Studies evaluating imaging and interventions

With the National Institute for Health and Care Excellence guidelines restricting ultrasound to infants less than 6 months of age, and both the National Institute for Health and Care Excellence and the American Academy of Pediatrics no longer recommending VCUG following an uncomplicated first febrile UTI, 39 studies applied the newer guidelines to a retrospective fully investigated cohort to determine what would have been missed, at least until a second febrile UTI. As expected, all studies determined that omitting a VCUG would have resulted in fewer cases of VUR being detected. This was deemed acceptable in 20 studies (13 medical [83–95], five surgical [96–100], two combined [101, 102]), with the newer guidelines, relying on US, considered able to detect most significant abnormalities including grade IV–V VUR, while often missing lesser grade II–III VUR with absent or mild dilatation, that would be found in the event of recurrent UTI, with savings in terms of reduced imaging costs and radiation exposure. In contrast, performance of a VCUG was deemed necessary in 19 studies (11 surgical [98, 103–112], six medical [113–118], two combined [119, 120]) with concern expressed that the abnormalities missed, in particular VUR of any grade, would place children at an indeterminate but possibly significant risk of morbidity.

Other studies by pediatricians were six in total and included two papers on compliance with the National Institute for Health and Care Excellence and revised American Academy of Pediatrics guidelines [121–123], two RCTs [2, 124], a meta-analysis of continuous antibiotic prophylaxis for prevention of scarring [125], and a study of scarring and cancer risk following VCUG [126]. Other publications by surgeons were four in total and included a retrospective assessment of UTI post-surgery, citing a febrile UTI incidence of 6.5% [127] whereas the only prospective RCT where children were maintained on continuous antibiotic prophylaxis post-surgery had a febrile UTI incidence of 21% [128], a study of surgical technique with recommendations on investigation and management [129], and a lower urinary tract dysfunction study [130].

Of the 58 studies that reported on surgical technique for correction of VUR, all but one [129] were excluded from this review, as no recommendations were made regarding investigation or management. The excluded papers are listed in Supplementary Material 2. Furthermore, only ten papers gave indications for surgery including breakthrough UTI despite antibiotic prophylaxis, deteriorating kidney function, and parental preference, while 44 were without indications for performing surgery. The 57 excluded papers represented the largest group of studies with an implicit assumption that VUR is a disease to be cured.

## Discussion

The literature search performed during the 11-year period from 2011, when the revised American Academy of Pediatrics guidelines on the investigation and management of a first febrile UTI in infancy were published, largely concurring with the earlier National Institute for Health and Care Excellence guidelines, demonstrated significant differences in approach between physicians and surgeons in terms of imaging, antibiotic prophylaxis, and surgery in the event of VUR detection. It should be clarified that this is not a systematic review or meta-analysis, which represent scholarly syntheses of evidence on a subject to inform healthcare decisions. While physicians have largely embraced evidence-based medicine, surgeons in many cases have not; thus, any systematic review or meta-analysis would potentially exclude large portions of the surgical literature and thus make any assessment of conflicting views between the two groups impossible.

An analysis of the papers identifies some indication of the justifications given by physicians and surgeons for the divergence of opinion. Physicians as a group, in line with the newer evidence-based guidelines, advocate less imaging and intervention, and are inclined to adopt a “watchful-waiting” approach, confident that any significant abnormality, grade IV–V VUR in particular, should be picked up following a second febrile UTI. In contrast, surgeons as a group are more likely to recommend imaging to detect VUR, with antibiotic prophylaxis and/or surgical correction if it is detected, concerned that any delay in diagnosis and treatment could place the child at risk of kidney damage. This divergence of approach between physicians and surgeons often confuses the family of the child, regarding the choice of how to best proceed with the diagnostic and therapeutic process.

Physicians have for the most part embraced evidence-based medicine following its inception at McMaster University in 1991 [131]. In 2007, the BMJ conducted an international poll to determine the most important medical milestones in healthcare. Evidence-based medicine came seventh, ahead of the computer and medical imaging [132]. Surgeons have been more tempered in accepting the evidence-based concept [133]. It would be easy to be dismissive of surgeons who do not practice evidence-based medicine; however, physicians had similar reservations initially [134]. Stirrat, who has previously published on the challenge of evaluating surgical procedures [135], explored the experiential nature of surgery and reasons for the slow uptake of evidence-based surgery. He acknowledges the benefits of evidence-based medicine but expresses concern regarding over-reliance on RCTs, and a lack of generalizability of evidence to individual patients. These remain valid concerns, particularly when there is absent, incomplete, or conflicting evidence as well as the recognition that RCTs determine net results in the groups studied, while the probabilities are not precisely transferable to all individuals within the groups [136]. These may be factors leading to less enthusiasm for the tenets of evidence-based medicine amongst surgeons. He also noted that surgeons often used historical controls, comparing the results of a new operation with those obtained using another procedure. This introduces serious bias due to the assumption that nothing has changed apart from the new procedure, with incorrect conclusions in 40–60% of such studies. Weil [137], in a commentary on the lack of RCTs in surgery, noted that “The case series remains a favored method of clinical investigation in surgery. Case series are easy to perform, require less resources in terms of personnel and funds, can be performed at a single center, and, for many surgeons, represent a means to illustrate their surgical method and skills.” This concern is particularly relevant to the large number of studies on surgical technique to correct VUR, where success rates in resolving VUR were paramount, with little or no discussion on the indications or outcomes.

Following the recognition by improved antenatal ultrasound of congenital abnormalities of the kidney and urinary tract as a major reason for extensive renal damage, along with RCTs demonstrating medical intervention (continuous antibiotic prophylaxis) to be largely ineffective in preventing febrile UTIs, with no effect on scarring [125], updated evidence-based guidelines in the medical literature have led to a marked reduction in the investigations performed, and treatment prescribed by physicians following a first febrile UTI [138] (Table 3). In contrast, many surgeons remain focused on VUR, as a disease to be investigated for and treated, in the absence of any prospective RCTs demonstrating benefit for their intervention, so much so that the largest group of studies in the surgical literature assess surgical techniques for correcting VUR without alluding to any indications for the procedures.

**Table 3:** Published guidelines for imaging following a first febrile urinary tract infection

Guideline	Ultrasound	VCUG	Late DMSA
NICE 2007 [139]	<6/12 **Yes** >6/12 atypical UTI	**No** unless abnormal US or atypical UTI	Atypical UTI
AAP 2011 [7]	**Yes**	**No** unless abnormal US	**No**
ISPN 2012 [140]	**Yes**	**No** unless abnormal US or risk factors	Abnormal US and/or VUR
CARI 2014 [141]	**No** unless absent antenatal US, atypical UTI, mass, poor stream, slow response	**No** unless recurrent febrile UTIs or US suggestive of posterior urethral valve	**No** unless reduced kidney function
Canadian 2014 [142]	**Yes**	**No** unless abnormal US suggestive of obstruction or high grade VUR	
EAU-ESPU 2015 [143]^a^	**Yes**	**Yes** consider after second febrile UTI in boys >1 year with option of a “top down” approach performing DMSA instead and VCUG if positive	**No** DMSA if VCUG positive or VCUG if DMSA positive
Urology Section AAP 2012 [74]^b^	**Yes**	**Yes**	**No**

^a^The EUA-ESPU guidelines were updated in 2021 (https://doi.org/10.1016/j.jpurol.2021.01.037), restricting a VCUG to those with an abnormal ultrasound or atypical UTI. The EUA-ESPU guidelines remain referenced in the article as these were the guidelines quoted by authors in the publications reviewed

^b^The Urology Section of the AAP published a dissenting view on the updated AAP guidelines regarding the investigation and management of a first febrile UTI, concerned that lack of a VCUG placed children with unrecognized VUR at risk of pyelonephritis and scarring

*AAP* American Academy of Pediatrics, *CARI* Caring for Australian and New Zealanders with Kidney Impairment, *EAU-ESPU* European Association of Urology – European Society of Pediatric Urology, *DMSA* ^99m^Tc-dimercaptosuccinic acid scan, *ISPN* Italian Society of Pediatric Nephrology, *NICE* National Institute for Health and Care Excellence, *US* ultrasound, *UTI* urinary tract infection, *VCUG* voiding cystourethrogram

This is not universally the case, however, with other surgeons stating that “vesicoureteral reflux is a phenotype not a disease” and is thus inconsequential [47]. Furthermore, RCTs in children with VUR that compared continuous antibiotic prophylaxis with surgical correction, demonstrated a low incidence of subsequent scarring with no significant difference between treatment arms, although no study has had a no-treatment control group [144–146].

An additional factor often raised is the mode of practice. Physicians and surgeons graduate from the same medical schools, after which their training diverges. Physicians focus on lifestyle counselling and medication where appropriate, often involving a long-term palliative rather than a curative approach, with the ability to re-assess management as newer information becomes available. Surgeons perform definitive procedures that historically were emergency interventions, such as appendectomy, with a predominance of elective procedures only developing in recent times. Regardless, given the invasive nature and irreversibility of operations, surgeons must have a strong belief in the curative nature of their interventions to convince patients, or the parents of patients, to consent. Are the differences phenotypic with different inherent traits determining the training path undertaken, as suggested in the BMJ paper [5], or are they the consequence of different approaches to training, as proposed by Stirrat [6]?

Comments such as “the literature obfuscates more than it clarifies” [82], “these guidelines do not reflect the real world” [129], and assertions that some guidelines (National Institute for Health and Care Excellence, American Academy of Pediatrics and Italian included) “are driven by economic and health care issues” [143], pervade the surgical literature. The admonition “You can’t handle the truth!” has even been applied to the pediatric community when they debate the need for continuous antibiotic prophylaxis in children following a UTI [82].

The 2008 National Institute for Health and Care Excellence evidence-based guidelines on UTI in infants and young children were the first to advocate reduced imaging and intervention [139]. Thereafter, published physician-instigated guidelines largely concurred with the National Institute for Health and Care Excellence, recommending additional imaging only when ultrasound was abnormal or in the few cases where recurrent febrile UTIs occurred [7, 140–142]. Otherwise, they promoted a “watchful waiting” approach, with less emphasis on VUR given the lack of evidence that intervention is of any clinical benefit, except possibly for high grade IV–V VUR, which accounts for <5% of cases and is likely to be detected in the event of a second febrile UTI.

The European Association of Urology - European Society for Paediatric Urology 2015 was the only guideline formulated by surgeons that addresses investigation and management of a first febrile UTI in children. It advocated VCUG to detect VUR [143] during the study period of this paper. The guideline has been recently updated, recommending VCUG following a first febrile UTI where an ultrasound was abnormal or the infection was due to an atypical organism [147]. The authors of the guideline did not reference any RCT and lament the low quality of evidence cited, acknowledging most of the recommendations are based on “panel and expert opinion” [148]. The relative lack of prospective well-designed studies involving elective surgical procedures (such as correction of VUR) is highlighted by data from major publicly-funded research bodies such as the UK’s National Institute for Health Research and Medical Research Council. Their combined spend on surgical research has been reported as <2% of the total research budget of £1.53 billion, even though 30% of National Health Service patients receive surgical care [149]. Similar funding levels are reported in other countries [137].

There appear to be two distinct directions in the literature on the investigation and management of febrile UTIs in children, one published predominantly by physicians in medical journals, and the other predominantly by surgeons in surgical journals. In undertaking this comparative study, we came to realize that, as physicians, almost all our reading has been in the medical literature, with the likelihood that surgeons may restrict their reading to the surgical literature. If we are to achieve consensus on the optimal management of conditions that cross boundaries between the physician and surgeon, as in the case of childhood febrile UTI, then improved collaboration in research as well as publications highlighting differences in points of view in both literatures is essential. Pediatric radiologists participate in multidisciplinary meetings and work closely with both pediatric nephrologists and surgeons. They may be in a good position to drive both research and consensus in the future.

How should the pediatric radiologist respond when asked to perform a VCUG in an otherwise normal child with a first febrile UTI? Firstly, the obvious disadvantages of the VCUG must be considered. Although a diagnostic study can be achieved with a relatively low radiation dose by using careful technique and modern equipment [150], in practice the range of doses is extremely wide [151]. It is worth noting that the European diagnostic reference level for dose-area product of 70 µGy·m^2^ for a child aged between one month and four years [150] corresponds to an effective dose of about 700 µSv, although this is at the upper end of a very wide range [151]. The risk of induced cancer from this exposure depends on a controversial conversion factor, but for the purposes of informed consent could reasonably be estimated at one in 5,000. Most VCUG procedures are uneventful, but severe complications have been reported, including fatal sepsis [152]. Finally, the psychological trauma of the procedure is difficult to quantify, but is probably significant, both for the patient and their carers [153, 154].

The risk of a child without other congenital abnormalities of the kidney and urinary tract developing chronic kidney disease as a result of repeated febrile UTIs associated with VUR is very low [3, 39, 155]. The shorter-term benefit of the possible prevention of further febrile UTIs by continuous antibiotic prophylaxis or a surgical intervention must be small, given that the probability of recurrence after a first febrile UTI is also known to be low [39]. One small non-randomized study showed no advantage in terms of quality of life in those children who underwent surgery over those managed non-surgically [156]. Studies with no control group are unlikely to provide useful data because the natural history of VUR is improvement through childhood. In the absence of a RCT of surgical versus non-surgical management of unselected patients found to have VUR after a first febrile UTI, there is no evidence that performing VCUG is of benefit in this context. Given that there are well-established risks of VCUG, it must be regarded as unwarranted. McAlister [152], writing nearly 50 years ago, gave sound and succinct advice: “Cystography is not a benign procedure. It can result in death and a host of complications. It should be undertaken only when its findings have a reasonable chance of altering patient management.”

Potential limitations of the approach used here include exclusion of papers published in languages other than English, and coverage of a limited, although recent, time period.

## Conclusion

There are two distinct directions in the literature regarding the investigation of an uncomplicated first febrile UTI in a child. In general, when presented with a first febrile UTI in a child, physicians recommended fewer investigations and less treatment, in contrast to surgeons who advocated extensive investigation and aggressive intervention in the event that imaging detects an abnormality. This has the potential to confuse the carers of affected children.

### Supplementary Information

Below is the link to the electronic supplementary material.

Supplementary file1 (DOCX 14 KB)

Supplementary file2 (DOCX 25 KB)

## Data Availability

The datasets generated and analyzed during the current study are available from the corresponding author on reasonable request.
