# Supplementary material for: Conflicting views of physicians and surgeons concerning pediatric urinary tract infection: a comparative review
Source: Pediatr Radiol. 2023 Sep 30;53(13):2651–61. doi: 10.1007/s00247-023-05771-x (PMC10698093; doi:10.1007/s00247-023-05771-x)
Supplement: Supplementary file 1 — Supplementary file1 (DOCX 14 KB) [file 247_2023_5771_MOESM1_ESM.docx]

**Appendix 1.**

Database: Ovid MEDLINE® and OVID Embase®

Search Strategy:

--------------------------------------------------------------------------------

1 Urinary tract infections/
2 exp Pyelonephritis/
3 UTI.tw.
4 pyelonephritis.tw.
5 (urinary tract adj5 infection$).tw.)
6 1 or 2 or 3 or 4 or 5
7 Child/
8 child$.tw.
9 p?ediatric$.tw.
10 7 or 8 or 9
11 Vesico-ureteral reflux/
12 (vesico adj2 ureteral).tw.
13 (vesico adj2 ureteric).tw.
14 VUR.tw.
15 11 or 12 or 13 or 14
16 Ultrasound.tw.
17 Ultrasonography.tw.
18 cystogram.tw.
19 (cysto adj2 urethrogram).tw.
20 (cysto adj2 urethrography).tw.
21 VCUG.tw.
22 MCUG.tw. (59)
23 16 or 17 or 18 or 19 or 20 or 21 or 22
24 Anti-bacterial agents/
25 Anti-infective agents/
26 antibiotic$.tw.
27 antimicrobial.tw.
28 24 or 25 or 26 or 27
29 prophylaxis.tw.
30 preventative.tw.
31 (long adj2 term).tw.
32 long-term.tw.
33 continuous.tw.
34 29 or 30 or 31 or 32 or 33
35 28 and 34

36 ureteroneocystostomy.tw.

37 (uretero adj2 neocystostomy).tw.

38 endoscopic.tw.

39 subureteral.tw.

40 subureteric.tw.

41 (re adj2 implantation).tw.

42 36 or 37 or 38 or 39 or 40 or 41

43 6 and 10 and 15

44 6 and 10 and 23

45 6 and 10 and 35

46 6 and 10 and 42

47 43 or 44 or 45 or 46 (4227)

48 limit 47 to yr="2011 - 2021" (1210)
