# Supplementary material for: Conflicting views of physicians and surgeons concerning pediatric urinary tract infection: a comparative review
Source: Pediatr Radiol. 2023 Sep 30;53(13):2651–61. doi: 10.1007/s00247-023-05771-x (PMC10698093; doi:10.1007/s00247-023-05771-x)
Supplement: Supplementary file 2 — Supplementary file2 (DOCX 25 KB) [file 247_2023_5771_MOESM2_ESM.docx]

1. Baek M, Kim KD. Current surgical management of vesicoureteral reflux. *Korean J Urol* 2013;54(11):732-7. doi: 10.4111/kju.2013.54.11.732 [published Online First: 20131106]

2. Deng T, Liu B, Luo L, et al. Robot-assisted laparoscopic versus open ureteral reimplantation for pediatric vesicoureteral reflux: a systematic review and meta-analysis. *World J Urol* 2018;36(5):819-28. doi: 10.1007/s00345-018-2194-x [published Online First: 20180127]

3. Van Batavia JP, Nees SN, Fast AM, et al. Outcomes of vesicoureteral reflux in children with non-neurogenic lower urinary tract dysfunction treated with dextranomer/hyaluronic acid copolymer (Deflux). *J Pediatr Urol* 2014;10(3):482-7. doi: 10.1016/j.jpurol.2013.10.017 [published Online First: 20131112]

4. Chertin B, Arafeh WA, Zeldin A, et al. Endoscopic correction of VUR using vantris as a new non-biodegradable tissue augmenting substance: three years of prospective follow-up. *Urology* 2013;82(1):201-4. doi: 10.1016/j.urology.2013.01.024 [published Online First: 20130307]

5. Yeoh JS, Greenfield SP, Adal AY, et al. The incidence of urinary tract infection after open anti-reflux surgery for primary vesicoureteral reflux: early and long-term follow up. *J Pediatr Urol* 2013;9(4):503-8. doi: 10.1016/j.jpurol.2012.05.006 [published Online First: 20120617]

6. Warchol S, Krzemien G, Szmigielska A, et al. Comparison of results of endoscopic correction of vesicoureteral reflux in children using two bulking substances: Dextranomer/hyaluronic acid copolymer (Deflux) versus polyacrylate-polyalcohol copolymer (Vantris). *J Pediatr Urol* 2016;12(4):256 e1-4. doi: 10.1016/j.jpurol.2016.04.006 [published Online First: 20160510]

7. Asgari SA, Asl AS, Safarinejad MR, et al. High success rate with new modified endoscopic treatment for high-grade VUR: A pilot study with preliminary report. *J Pediatr Urol* 2016;12(2):100 e1-4. doi: 10.1016/j.jpurol.2015.07.013 [published Online First: 20150925]

8. Ure I, Gurocak S, Tan O, et al. Subureteral Injection with Small-Size Dextranomer/Hyaluronic Acid Copolymer: Is It Really Efficient? *Biomed Res Int* 2016;2016:2168753. doi: 10.1155/2016/2168753 [published Online First: 20161226]

9. Javali T, Pathade A, Nagaraj HK. Laparoscopic extravesical detrusorraphy, a minimally invasive treatment option for vesicoureteral reflux: a single centre experience. *J Pediatr Urol* 2015;11(2):88 e1-6. doi: 10.1016/j.jpurol.2015.01.007 [published Online First: 20150305]

10. Dogan HS, Bozaci AC, Ozdemir B, et al. Ureteroneocystostomy in primary vesicoureteral reflux: critical retrospective analysis of factors affecting the postoperative urinary tract infection rates. *Int Braz J Urol* 2014;40(4):539-45. doi: 10.1590/S1677-5538.IBJU.2014.04.14

11. Corbetta JP, Bortagaray JI, Weller S, et al. The use of polyacrylate-polyalcohol copolymer hydrogel in the endoscopic treatment of primary vesicoureteral reflux in children. *J Pediatr Surg* 2015;50(3):485-8. doi: 10.1016/j.jpedsurg.2014.07.001 [published Online First: 20140805]

12. Arlen AM, Broderick KM, Huen KH, et al. Temporal pattern of vesicoureteral reflux on voiding cystourethrogram correlates with dynamic endoscopic hydrodistention grade of ureteral orifice. *J Urol* 2014;192(5):1503-7. doi: 10.1016/j.juro.2014.05.024 [published Online First: 20140514]

13. Arlen AM, Scherz HC, Filimon E, et al. Is routine voiding cystourethrogram necessary following double hit for primary vesicoureteral reflux? *J Pediatr Urol* 2015;11(1):40 e1-5. doi: 10.1016/j.jpurol.2014.11.011 [published Online First: 20150130]

14. Grimsby GM, Dwyer ME, Jacobs MA, et al. Multi-institutional review of outcomes of robot-assisted laparoscopic extravesical ureteral reimplantation. *J Urol* 2015;193(5 Suppl):1791-5. doi: 10.1016/j.juro.2014.07.128 [published Online First: 20141007]

15. Sharifiaghdas F, Tajalli F, Otukesh H, et al. Endoscopic correction of primary VUR by using polyacrylate polyalcohol copolymer (Vantris) in young girls: 2-year follow-up. *J Pediatr Urol* 2014;10(6):1032-6. doi: 10.1016/j.jpurol.2014.02.016 [published Online First: 20140405]

16. Basiri A, Otookesh H, Simforoosh N, et al. Does pre-transplantation antireflux surgery eliminate post-renal transplantation pyelonephritis in children? *J Urol* 2006;175(4):1490-2. doi: 10.1016/S0022-5347(05)00670-1 [published Online First: 2006/03/07]

17. Choi W, Nam W, Lee C, et al. Long-term Outcomes of Endoscopic Anti-reflux Surgery in Pediatric Patients with Vesicoureteral Reflux: Urinary Tract Infection, Renal Scarring, and Predictive Factors for Success. *J Korean Med Sci* 2018;33(38):e240. doi: 10.3346/jkms.2018.33.e240 [published Online First: 20180808]

18. Moore K, Bolduc S. Prospective study of polydimethylsiloxane vs dextranomer/hyaluronic acid injection for treatment of vesicoureteral reflux. *J Urol* 2014;192(6):1794-9. doi: 10.1016/j.juro.2014.05.116 [published Online First: 20140610]

19. Hayashi Y, Mizuno K, Kurokawa S, et al. Extravesical robot-assisted laparoscopic ureteral reimplantation for vesicoureteral reflux: initial experience in Japan with the ureteral advancement technique. *Int J Urol* 2014;21(10):1016-21. doi: 10.1111/iju.12483 [published Online First: 20140520]

20. Dangle PP, Shah A, Gundeti MS. Robot-assisted laparoscopic ureteric reimplantation: extravesical technique. *BJU Int* 2014;114(4):630-2. doi: 10.1111/bju.12813 [published Online First: 20140814]

21. Mohamed EA, Shehata FH, Abdelbaset EA, et al. Longterm outcome of Macroplatique injection for treatment of vesicoureteral reflux in children. *Afr J Paediatr Surg* 2014;11(2):174-8. doi: 10.4103/0189-6725.132829

22. Tanhaeivash R, Kajbafzadeh AM, Zeinoddini A, et al. Combination of calcium hydroxyapatite and autologous blood for endoscopic treatment of vesicoureteral reflux in children. *Int Urol Nephrol* 2014;46(7):1263-8. doi: 10.1007/s11255-014-0659-4 [published Online First: 20140220]

23. Sencan A, Ucan B, Evciler H, et al. Early results of endoscopic treatment of vesicoureteral reflux with polyacrylate polyalcohol copolymer. *Urol Int* 2014;92(2):219-22. doi: 10.1159/000354879 [published Online First: 20131205]

24. Sencan A, Yildirim H, Ozkan KU, et al. Open ureteroneocystostomy after failed endoscopic injection with three different bulking agents for the treatment of vesicoureteral reflux. *J Pediatr Surg* 2014;49(11):1652-5. doi: 10.1016/j.jpedsurg.2014.04.012 [published Online First: 20140602]

25. Sencan A, Yildirim H, Ozkan KU, et al. Late ureteral obstruction after endoscopic treatment of vesicoureteral reflux with polyacrylate polyalcohol copolymer. *Urology* 2014;84(5):1188-93. doi: 10.1016/j.urology.2014.07.030 [published Online First: 20141024]

26. Fotso Kamdem A, Galli G, Aubert D. Long-term incidence of febrile UTI after DxHA treatment of VUR. *J Pediatr Urol* 2014;10(1):56-61. doi: 10.1016/j.jpurol.2013.06.002 [published Online First: 20130626]

27. Kim KH, Lee YS, Im YJ, et al. A modified technique for ureteral reimplantation: intravesical detrusorrhaphy. *J Pediatr Surg* 2013;48(8):1813-8. doi: 10.1016/j.jpedsurg.2013.05.017

28. Watters ST, Sung J, Skoog SJ. Endoscopic treatment for vesicoureteral reflux: how important is technique? *J Pediatr Urol* 2013;9(6 Pt B):1192-7. doi: 10.1016/j.jpurol.2013.05.002 [published Online First: 20130612]

29. Garcia-Aparicio L, Rodo J, Palazon P, et al. Acute and delayed vesicoureteral obstruction after endoscopic treatment of primary vesicoureteral reflux with dextranomer/hyaluronic acid copolymer: why and how to manage. *J Pediatr Urol* 2013;9(4):493-7. doi: 10.1016/j.jpurol.2013.02.007 [published Online First: 20130316]

30. Cloutier J, Blais AS, Moore K, et al. Prospective study using a new bulking agent for the treatment of vesicoureteral reflux: polyacrylamide hydrogel. *J Urol* 2013;190(3):1034-7. doi: 10.1016/j.juro.2013.03.071 [published Online First: 20130326]

31. Ramsay S, Blais AS, Morin F, et al. Polyacrylamide Hydrogel as a Bulking Agent for the Endoscopic Treatment of Vesicoureteral Reflux: Long-Term Results and Safety. *J Urol* 2017;197(3 Pt 2):963-67. doi: 10.1016/j.juro.2016.08.093 [published Online First: 20160826]

32. Kajbafzadeh AM, Tourchi A. Usefulness of concomitant autologous blood and dextranomer/hyaluronic acid copolymer injection to correct vesicoureteral reflux. *J Urol* 2012;188(3):948-52. doi: 10.1016/j.juro.2012.04.119 [published Online First: 20120721]

33. Kajbafzadeh AM, Tourchi A, Aryan Z. Factors that impact the outcome of endoscopic correction of vesicoureteral reflux: a multivariate analysis. *Int Urol Nephrol* 2013;45(1):1-9. doi: 10.1007/s11255-012-0327-5 [published Online First: 20121117]

34. Riquelme M, Lopez M, Landa S, et al. Laparoscopic extravesical ureteral reimplantation (LEVUR): a multicenter experience with 95 cases. *Eur J Pediatr Surg* 2013;23(2):143-7. doi: 10.1055/s-0032-1329708 [published Online First: 20121119]

35. Puri P, Kutasy B, Colhoun E, et al. Single center experience with endoscopic subureteral dextranomer/hyaluronic acid injection as first line treatment in 1,551 children with intermediate and high grade vesicoureteral reflux. *J Urol* 2012;188(4 Suppl):1485-9. doi: 10.1016/j.juro.2012.02.023 [published Online First: 20120817]

36. Kasturi S, Sehgal SS, Christman MS, et al. Prospective long-term analysis of nerve-sparing extravesical robotic-assisted laparoscopic ureteral reimplantation. *Urology* 2012;79(3):680-3. doi: 10.1016/j.urology.2011.10.052 [published Online First: 20111223]

37. Bayne AP, Shoss JM, Starke NR, et al. Single-center experience with pediatric laparoscopic extravesical reimplantation: safe and effective in simple and complex anatomy. *J Laparoendosc Adv Surg Tech A* 2012;22(1):102-6. doi: 10.1089/lap.2011.0299 [published Online First: 20111213]

38. Kalisvaart JF, Scherz HC, Cuda S, et al. Intermediate to long-term follow-up indicates low risk of recurrence after Double HIT endoscopic treatment for primary vesico-ureteral reflux. *J Pediatr Urol* 2012;8(4):359-65. doi: 10.1016/j.jpurol.2011.07.006 [published Online First: 20110804]

39. Chalmers D, Herbst K, Kim C. Robotic-assisted laparoscopic extravesical ureteral reimplantation: an initial experience. *J Pediatr Urol* 2012;8(3):268-71. doi: 10.1016/j.jpurol.2011.04.006 [published Online First: 20110608]

40. Kaye JD, Srinivasan AK, Delaney C, et al. Clinical and radiographic results of endoscopic injection for vesicoureteral reflux: defining measures of success. *J Pediatr Urol* 2012;8(3):297-303. doi: 10.1016/j.jpurol.2011.02.006 [published Online First: 20110504]

41. Chung KLY, Sihoe J, Liu K, et al. Surgical Outcome Analysis of Pneumovesicoscopic Ureteral Reimplantation and Endoscopic Dextranomer/Hyaluronic Acid Injection for Primary Vesicoureteral Reflux in Children: A Multicenter 12-Year Review. *J Laparoendosc Adv Surg Tech A* 2018;28(3):348-53. doi: 10.1089/lap.2017.0281 [published Online First: 20171222]

42. Dogan HS, Altan M, Citamak B, et al. Factors affecting the success of endoscopic treatment of vesicoureteral reflux and comparison of two dextranomer based bulking agents: does bulking substance matter? *J Pediatr Urol* 2015;11(2):90 e1-5. doi: 10.1016/j.jpurol.2014.12.009 [published Online First: 20150302]

43. Esposito C, Masieri L, Steyaert H, et al. Robot-assisted extravesical ureteral reimplantation (revur) for unilateral vesico-ureteral reflux in children: results of a multicentric international survey. *World J Urol* 2018;36(3):481-88. doi: 10.1007/s00345-017-2155-9 [published Online First: 20171216]

44. Friedmacher F, Colhoun E, Puri P. Endoscopic Injection of Dextranomer/Hyaluronic Acid as First Line Treatment in 851 Consecutive Children with High Grade Vesicoureteral Reflux: Efficacy and Long-Term Results. *J Urol* 2018;200(3):650-55. doi: 10.1016/j.juro.2018.03.074 [published Online First: 20180315]

45. Garcia-Aparicio L, Blazquez-Gomez E, Martin O, et al. Randomized clinical trial between polyacrylate-polyalcohol copolymer (PPC) and dextranomer-hyaluronic acid copolymer (Dx/HA) as bulking agents for endoscopic treatment of primary vesicoureteral reflux (VUR). *World J Urol* 2018;36(10):1651-56. doi: 10.1007/s00345-018-2314-7 [published Online First: 20180503]

46. Garcia-Aparicio L, Rovira J, Blazquez-Gomez E, et al. Randomized clinical trial comparing endoscopic treatment with dextranomer hyaluronic acid copolymer and Cohen's ureteral reimplantation for vesicoureteral reflux: long-term results. *J Pediatr Urol* 2013;9(4):483-7. doi: 10.1016/j.jpurol.2013.03.003 [published Online First: 20130418]

47. Gundeti MS, Boysen WR, Shah A. Robot-assisted Laparoscopic Extravesical Ureteral Reimplantation: Technique Modifications Contribute to Optimized Outcomes. *Eur Urol* 2016;70(5):818-23. doi: 10.1016/j.eururo.2016.02.065 [published Online First: 20160330]

48. Hacker FM, Frech-Dorfler M, von Rotz M, et al. Endoscopic hyaluronic acid/dextranomer gel implantation is effective as first-line treatment of vesicoureteral reflux (VUR) in children: a single centre experience. *Eur J Pediatr Surg* 2011;21(5):299-303. doi: 10.1055/s-0031-1279700 [published Online First: 20110617]

49. Harper L, Paillet P, Minvielle T, et al. Long-Term (>10 Years) Results After Endoscopic Injection Therapy for Vesicoureteral Reflux. *J Laparoendosc Adv Surg Tech A* 2018;28(11):1408-11. doi: 10.1089/lap.2018.0035 [published Online First: 20180723]

50. Ozkuvanci U, Donmez MI, Ozgor F, et al. Durasphere(R) EXP: a non-biodegradable agent for treatment of primary Vesico-Ureteral reflux in children. *Int Braz J Urol* 2018;44(3):585-90. doi: 10.1590/S1677-5538.IBJU.2017.0514

51. Stredele RJ, Dietz HG, Stehr M. Long-term results of endoscopic treatment of vesicoureteral reflux in children: comparison of different bulking agents. *J Pediatr Urol* 2013;9(1):71-6. doi: 10.1016/j.jpurol.2011.12.004 [published Online First: 20111231]

52. Tekin A, Yagmur I, Tiryaki S, et al. Changing bulking agent may require change in injection volume for endoscopic treatment of vesicoureteral reflux. *Int Braz J Urol* 2018;44(6):1194-99. doi: 10.1590/S1677-5538.IBJU.2018.0033

53. Blais AS, Morin F, Cloutier J, et al. Efficacy of dextranomer hyaluronic acid and polyacrylamide hydrogel in endoscopic treatment of vesicoureteral reflux: A comparative study. *Can Urol Assoc J* 2015;9(5-6):202-6. doi: 10.5489/cuaj.2964

54. Dwyer ME, Husmann DA, Rathbun SR, et al. Febrile urinary tract infections after ureteroneocystostomy and subureteral injection of dextranomer/hyaluronic acid for vesicoureteral reflux--do choice of procedure and success matter? *J Urol* 2013;189(1):275-82. doi: 10.1016/j.juro.2012.09.011 [published Online First: 20121120]

55. Esposito C, Escolino M, Lopez M, et al. Surgical Management of Pediatric Vesicoureteral Reflux: A Comparative Study Between Endoscopic, Laparoscopic, and Open Surgery. *J Laparoendosc Adv Surg Tech A* 2016;26(7):574-80. doi: 10.1089/lap.2016.0055 [published Online First: 20160610]

56. Kocherov S, Ulman I, Nikolaev S, et al. Multicenter survey of endoscopic treatment of vesicoureteral reflux using polyacrylate-polyalcohol bulking copolymer (Vantris). *Urology* 2014;84(3):689-93. doi: 10.1016/j.urology.2014.04.033

57. Lee JN, Lee SM, Ha YS, et al. VUR timing on VCUG as a predictive factor of VUR resolution after endoscopic therapy. *J Pediatr Urol* 2016;12(4):255 e1-6. doi: 10.1016/j.jpurol.2016.04.002 [published Online First: 20160506]
